# Sentinel lymph node biopsy provides better regional control than observation in early stage maxillary squamous cell carcinoma

**DOI:** 10.3389/fonc.2025.1623502

**Published:** 2025-06-24

**Authors:** Yannan Wang, Xu Zhang, Junhui Yuan, Zhengquan Zhou, Wei Du, Fei Liu, Jing Wang

**Affiliations:** ^1^ Department of Stomatology, The First Affiliated Hospital of Zhengzhou University, Zhengzhou, China; ^2^ ^C^ Department of Head and Neck, The Affiliated Cancer Hospital of Zhengzhou University & Henanancer Hospital, Zhengzhou, China; ^3^ Department of Radiology, The Affiliated Cancer Hospital of Zhengzhou University & Henan Cancer Hospital, Zhengzhou, China

**Keywords:** sentinel lymph node biopsy, neck management, maxillary squamous cell carcinoma, propensity score matching, prognosis

## Abstract

**Background:**

An observational approach in the management of the cervical region in instances of maxillary squamous cell carcinoma (SCC) results in appreciable treatment failure, necessitating a more efficacious neck intervention. Our objective was to compare the efficacy of sentinel lymph node biopsy (SLNB) versus observation regarding regional control (RC) in patients diagnosed with cT1/2N0 maxillary SCC.

**Methods:**

Patients who underwent SLNB or observation for neck management in primary cT1/2N0 maxillary SCC were retrospectively enrolled. SLNB was performed one day before surgery using 99mTc-nanocolloid, with the decision to proceed with neck dissection guided by intraoperative sentinel lymph node pathology. Primary outcome variable was 5-year RC. Impact of neck management on RC was evaluated using Kaplan-Meier survival analysis and Cox proportional hazards modeling.

**Results:**

A total of 145 patients were included, with SLNB performed in 46 necks and observation applied to 99 cases. Sentinel lymph nodes were successfully identified in all instances. In comparison, regional recurrence was observed in eight patients within the SLNB cohort, of whom six had previously exhibited a metastatic sentinel lymph node, as opposed to 21 patients in the observational group. In the Cox model, SLNB was associated with a relative 28% reduction in the risk of neck failure compared to observation (p=0.021). Occult lymph node metastasis was more prevalent in tumors possessing a diameter exceeding 2cm or a depth of invasion surpassing 4mm, subgroup analysis revealed that the positive impact of SLNB on outcomes became statistically significant only for tumors larger than 2.0 cm (p=0.041, hazard ratio=0.23, 95% confidence interval: 0.05-0.98) or with a depth of invasion greater than 4.0 mm (p=0.042, hazard ratio=0.22, 95% confidence interval: 0.02-0.95).

**Conclusion:**

SLNB demonstrated superior RC compared to observation in patients diagnosed with cT1/2N0 maxillary SCC. These results support reconsidering SLNB as a standard approach among those with tumors exceeding 2.0 cm in size or a depth of invasion surpassing 4.0 mm.

## Introduction

Oral squamous cell carcinoma (SCC) is the predominant neoplasm in the head and neck region, and complete excision acts as the preferred treatment modality (1). The prognosis of this disease is significantly influenced by the status of the cervical lymph nodes (LNs), with the presence of even a single positive LN leading to an apparent reduction in survival rates. (2) Given the critical role of LN involvement in prognosis, precise neck dissection has become a focal point of investigation, particularly in advanced-stage cases (cT3/4). However, the role of neck dissection in cases classified as cT1/2 remains a subject of ongoing discussion and investigation.

In a groundbreaking trial conducted in 2015, the positive impact of elective neck dissection was unequivocally demonstrated, showing a statistically significant improvement in both 3-year overall survival and disease-free survival rates compared to observation followed by therapeutic neck dissection (3). Consequently, elective neck dissection approach is preferred in managing early stage oral SCC. However, occult metastasis could be omitted by preoperative imaging analysis and occurs in 20%-30% of these early stage tumors (4, 5), resulting in a substantial number of patients undergoing unnecessary treatment. Two trials have demonstrated that SLNB is both feasible and well-tolerated (6, 7). Subsequent studies from multiple institutions have shown that SLNB can achieve clinical outcomes comparable to elective neck dissection in certain cases (8–10). However, it is important to note that existing studies often exclude cases localized in the upper gingiva and hard palate, where SCC has a lower propensity for metastasis than those arising in tongue and the mouth floor. This exclusion underscores the need for further investigation into whether SLNB is an effective alternative for these types of cases.

Hence, the purpose of this study was to compare the efficacy of SLNB versus observation in terms of regional control (RC) and disease-specific survival (DSS) among patients diagnosed with cT1/2N0 maxillary SCC referring to early-stage tumors without clinical evidence of LN involvement.

## Patients and methods

### Ethical considerations

This study was approved by The Affiliated First Hospital of Zhengzhou University Institutional Research Committee. All procedures involving human participants were conducted according to the principles of the Declaration of Helsinki.

### Patient selection

**Figure 1:**
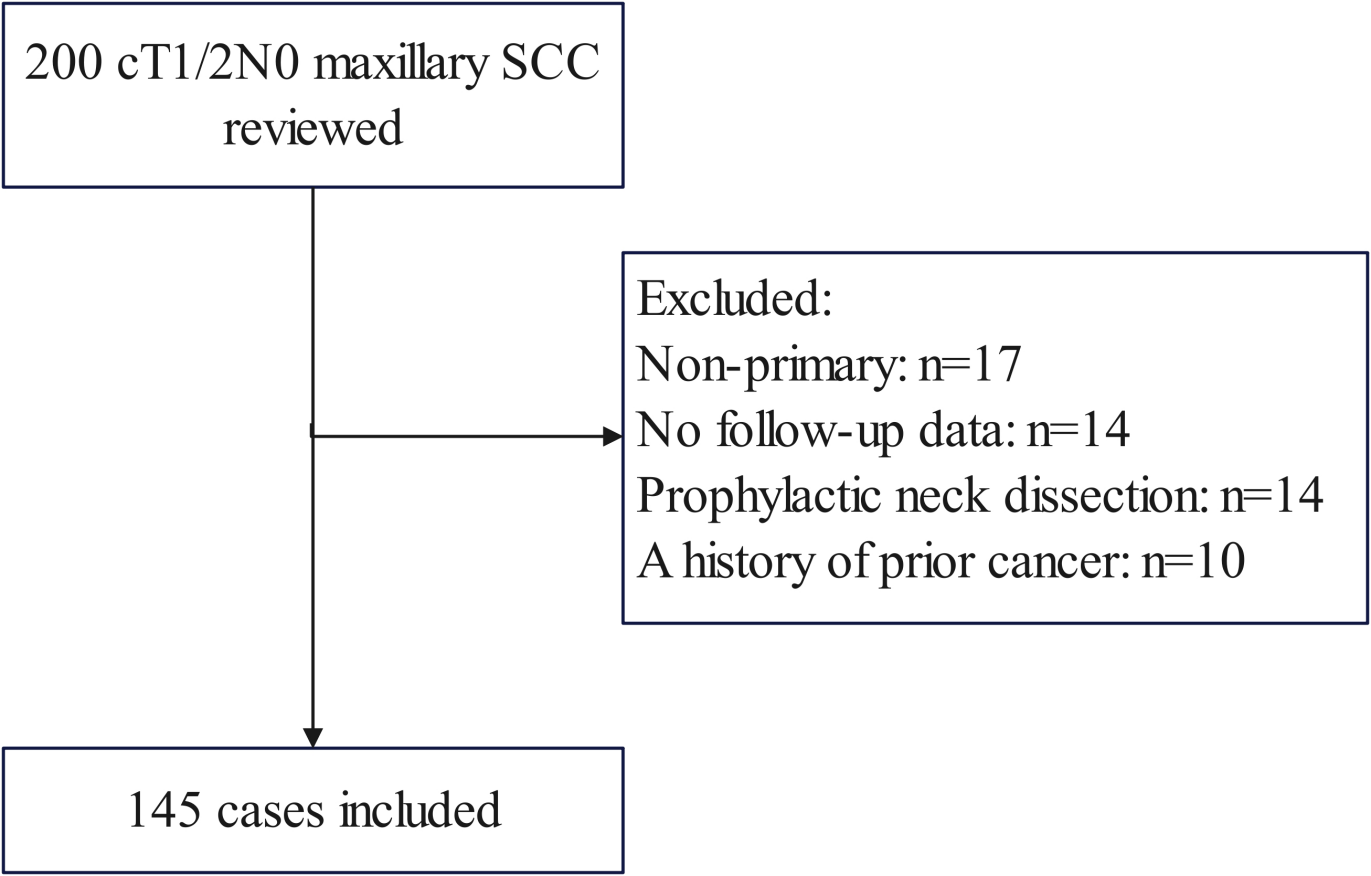
Patient inclusion and exclusion flow diagram. Among 200 initially identified patients with cT1/2N0 maxillary squamous cell carcinoma (SCC), 55 cases were excluded for the following reasons: non-primary tumors (n=17), lack of follow-up data (n=14), prophylactic neck dissection (n=14), and history of prior cancer (n=10).

To address the research purpose, the investigators designed and implemented a comprehensive review of medical records pertaining to patients who underwent surgical treatment for maxillary SCC during the period from January 2014 to December 2022. These criteria included: confirmation that the disease was primary and previously untreated, with the primary site located in the upper gingiva or hard palate; utilization of SLNB or observation as the neck management approach; tumor stage was categorized as T1/2 determined by preoperative clinical and imaging assessment based on the 8^th^ AJCC classification, with no clinical evidence of positive LNs. Exclusion criteria encompassed patients with a history of previous cancer (n=10), those lacking follow-up data (n=14), or individuals who had undergone prophylactic neck dissection (n=14) (**Figure 1**). We meticulously curated data on patient demographics, pathology reports, treatment modalities employed, and subsequent follow-up information via electronic health records.

### Variable definition

**Figure 2:**
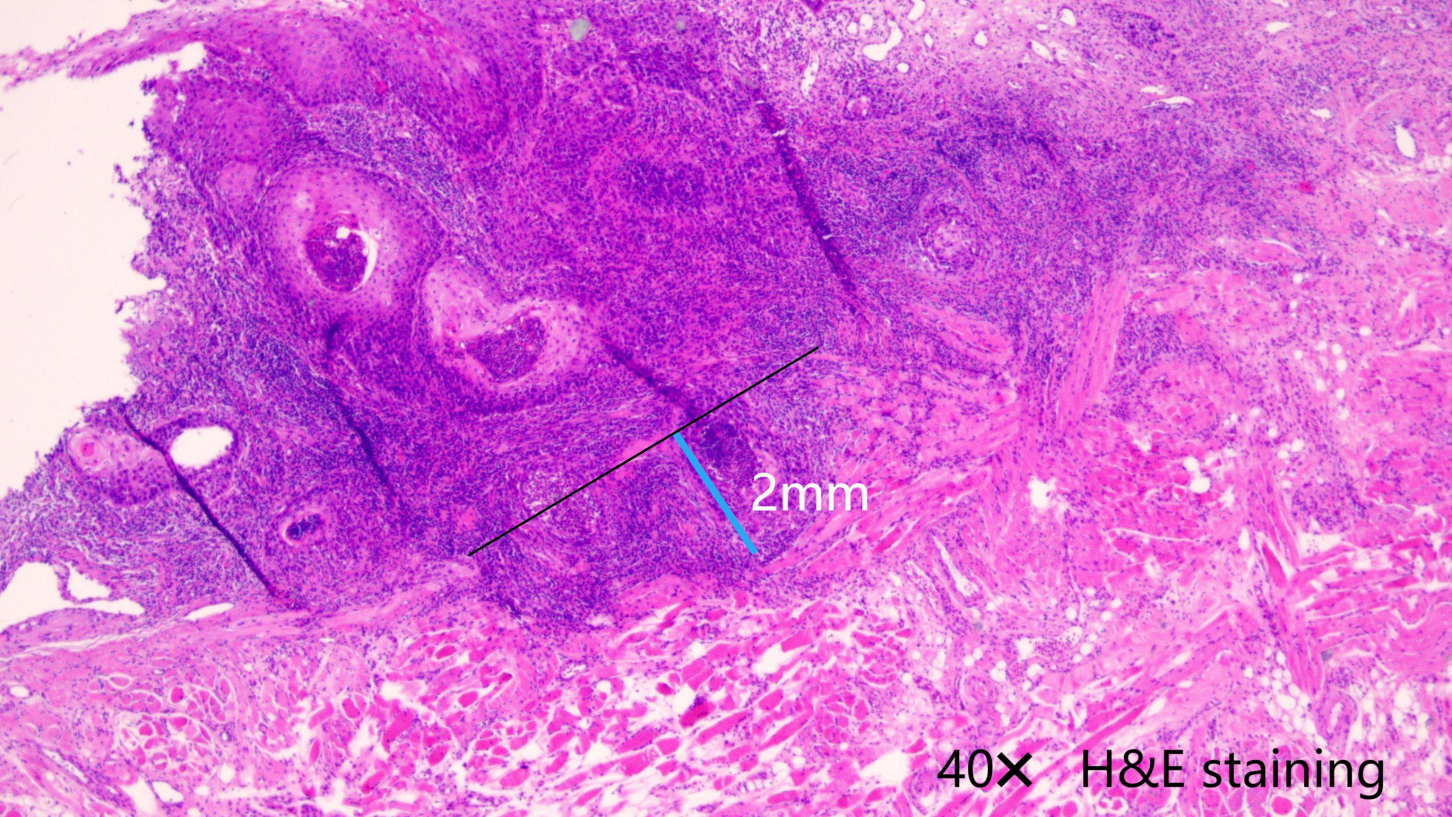
The depth of invasion was measured as the perpendicular distance from the basement membrane (black line) of the adjacent normal epithelium to the deepest point of tumor invasion (blue line), using 40× light microscopy on H&E-stained sections.

A smoker was an individual who had smoked at least 100 cigarettes in his or her lifetime or who now smoked every day (11). Drinkers were defined as individuals who consumed at least one standard alcoholic beverage per day (≥14 grams of pure alcohol/day) for a minimum of one year (12). Tumor classification as cT1/2 was based on the 8th AJCC system, while cN0 status was determined through ultrasound and contrast-enhanced CT imaging, confirming the absence of clinically positive LNs. Histologic differentiation categories encompassed well, intermediate, and poor. Perineural invasion (PNI) was defined as cancer cell infiltration along nerve pathways, with or without direct neural destruction, and lymphovascular invasion (LVI) was defined as the presence of tumor emboli within endothelial-lined lymphatic or blood vessels, as confirmed by histopathological analysis (13). The DOI was determined histologically as the perpendicular measurement from the basement membrane of the adjacent normal epithelium to the deepest point of tumor invasion using light microscopy on H&E-stained sections (**Figure 2**) (14).

The primary outcome was 5-year RC, while the secondary outcome was 5-year DSS. RC was defined as the time from surgery to the first occurrence of neck recurrence or last follow-up visit. DSS was defined as the time from surgery to cancer-related death or last follow-up visit.

### Treatment

One day before surgery, ^99m^Tc-nanocolloid was injected in the submucosal layer around the circumference of the primary tumor via direct visual placement. Dynamic lymphoscintigraphy was done in the anterior and lateral views followed by a single photon emission computed tomography examination one hour later. Precise location of the sentinel LN was delineated on the overlying skin using methylene blue staining (6–9). In cases where the sentinel LN was not visualized through lymphoscintigraphy or detected using a gamma probe, a strategy of observation was typically recommended, and these patients were evaluated in observation group.

Primary tumors underwent resection with a minimum margin of 1cm, and this margin width was determined intraoperatively by macroscopic measurement using a sterile ruler, ensuring a visible and palpable tumor-free margin around the primary tumor. While sentinel LNs were excised employing a transcervical approach. Frozen section analysis was carried out in all patients to ensure a negative margin and assess the sentinel LN metastatic status. Should occult metastasis be confirmed, dissection of levels I to IV/V was performed based on the surgeon’s experience, positive sentinel LN location, tumor size, and DOI. Adjuvant radiotherapy was administered in cases featuring PNI, LVI, poor differentiation, or other unfavorable pathological characteristics. Adjuvant chemotherapy with cisplatin or carboplatin plus other agents was administered for extranodal extension or positive margins, per institutional protocols.

### Statistic analysis

Missing rates among the variables were 14.5% for PNI, 13.8% for LVI, and 9.7% for pathologic grade. Missing data patterns among PNI, LVI, and pathologic grade were deemed not missing completely at random clinically and methodologically (15). On one hand, missing data were concentrated in older records where documentation standards evolved, suggesting missing at random mechanisms tied to temporal practice changes. On the other hand, cross-tabulation of missingness against covariates revealed no systematic bias in absence patterns relative to outcomes. By employing multiple imputation rather than deletion, we mitigated potential bias even under missing at random (16).

Descriptive statistics were used to summarize the dataset. For normally distributed continuous variables, mean and standard deviation were reported. For skewed data, median and range were used. Categorical variables were expressed as frequencies and percentages.

The clinicopathological variables between SLNB and observation groups were compared using the Chi-square test. Impact of different neck managements on RC and DSS was assessed using univariate analysis and Cox model with presentation via hazard ratio (HR) and 95% confidence interval (CI) in the whole cohort. These independent prognostic factors were included in a propensity score matching (PSM) model, and a 1:1 matching was performed to appropriately adjust for confounding factors between the two groups. Then, a second analysis of the influence was conducted in the matched population, and also the influence was assessed in subgroups stratified by DOI and tumor size. Statistical analyses were performed using R software (version 3.4.3; R Foundation for Statistical Computing, Vienna, Austria), considering a statistical significance level of p < 0.05.

## Results

### Baseline data

A total of 145 patients were enrolled in this study, comprising 99 (68.3%) males and 46 (31.7%) females, with a mean age of 52 ± 17 years. Among the participants, 73 (50.3%) were identified as smokers, while 39 (26.9%) were classified as drinkers. In terms of clinical tumor staging, 62 (42.8%) patients fell into the T1 category, whereas 83 (57.2%) patients were categorized as T2. Notably owing to DOI evaluation, three T1 tumors were subsequently reclassified as T2 during permanent pathology assessment, whereas ten T2 tumors were downgraded to T1. Midline involvement was observed in 24 patients. PNI was observed in 38 (26%) patients, while LVI was present in 31 (21%) patients. Pathologic grading revealed that 47 (32.4%) patients had well-differentiated tumors, 63 (43.4%) had intermediate differentiation, and 35 (24.1%) had poor differentiation. The mean DOI was determined to be 4.9 ± 2.0mm. All patients achieved negative surgical margins. Those treated with SLNB displayed a higher prevalence of poor differentiation and PNI, as depicted in **Table 1**.

**Table 1:** Clinicopathological variables between sentinel lymph node biopsy (SLNB) and conservative treatment groups.

Variable	SLNB (n=46)	Conservative treatment (n=99)	P
Age			
<50	21	50	
≥50	25	49	0.586
Sex			
Male	32	67	
Female	14	32	0.820
Smoker	20	53	0.260
Drinker	11	28	0.581
Pathologic tumor stage			
T1	17	52	
T2	29	47	0.081
Midline involvement	10	14	0.252
PNI^	20	18	0.001
LVI*	13	18	0.168
Pathologic grade			
Well	8	39	
Intermediate	20	43	
Poor	18	17	0.004

^PNI, perineural invasion; *LVI, lymphovascular invasion.

The SLNB group consisted of 46 patients, and sentinel LNs were successfully obtained in 100% using lymphoscintigraphy and/or gamma probe. The mean and median number of dissected sentinel LNs were calculated as 2 ± 1 and 2, respectively, with a range of 1 to 6 LNs. Among the dissected LNs, the most common location was identified as level I (n=30), followed by level II (n=13), level III (n=7), level IV (n=6), and level V (n=1). Bilateral sentinel lymph node (SLN) involvement was identified in only 2 patients, exclusively in cases with midline tumor crossover. In these patients, SLNs were located bilaterally in levels I and II, reflecting the expected lymphatic drainage pattern of midline tumors, where lymphatic spread can occur to both sides of the neck. The data on midline involvement are presented to highlight its role as a predictor of bilateral nodal spread, which is critical for surgical planning; no instances were noted in solo contralateral or parapharyngeal or retropharyngeal locations. The distribution pattern of sentinel LNs revealed that 35 patients had LNs identified in a single level, whereas 11 patients exhibited sentinel LNs in two different levels. Among the patients who underwent SLNB, eight had positive results. Of these, seven exhibited level I metastasis, while one showed level II metastasis. All eight patients subsequently underwent therapeutic neck dissection, with no evidence of extranodal extension identified. Additionally, adjuvant radiotherapy was administered to 20 patients with a mean dose of 56 ± 6Gy. Conversely, a cohort of 99 patients underwent a conservative approach. Within this group, 34 individuals received adjuvant radiotherapy, encompassing the primary site and partial levels I and II. The mean radiation dose administered to these patients was 52 ± 10Gy.

During a follow-up with median time of 2.9 (range: 0.3-7.9) years, there was no distant metastasis, within the SLNB group, a total of eight patients experienced regional recurrence, with two of these cases also manifesting local recurrence, remarkably, two of these patients had previously received a negative SLNB result due to technique limitation. In an effort to address the recurrent disease, salvage radical neck dissection were undertaken in two patients. Unfortunately, six cases died. In the observation group, two patients developed isolated local recurrence, while eighteen experienced regional neck metastasis. Locoregional recurrence was observed in three patients (**Table 2**). Salvaged surgery was performed in nine individuals, and a total of fifteen patients lost their life.

**Table 2:** Failure pattern in patients undergoing sentinel lymph node biopsy (SLNB) or observation.

Failure site	SLNB	Observation
Local	2	5
Regional		
Level I	4	14
Level II	3	8
Level III	2	5
Level IV	2	2
Level V	0	1

### Univariate and multivariable analysis

In order to examine the potential impact of clinicopathological factors on RC and DSS, an initial assessment of these factors was conducted through univariate analysis. SLNB did not provide a protective effect when compared to observation alone (p=0.091 for RC and p=0.101 for DSS). This comprehensive analysis revealed a statistically significant association between tumor size (p=0.016 for RC and p=0.012 for DSS), DOI (p=0.009 for RC and p=0.011 for DSS), and pathological grade (p=0.012 for RC and p=0.001 for DSS) with both RC and DSS, while PNI was found to be significantly related to RC (p=0.028) rather than DSS (p=0.135) (as shown in **Table 3**). Consequently, these influential factors were subjected to further scrutiny using a Cox model. In further multivariate analysis, tumor size (p=0.014, HR: 2.63, 95%CI: 1.21-5.85; p=0.005, HR: 3.04, 95%CI: 1.45-8.14), DOI (p=0.002, HR: 3.19, 95%CI: 1.62-9.87; p=0.003, HR: 4.35, 95%CI: 1.58-10.52), and poor differentiation (p=0.024, HR: 3.16, 95%CI: 2.14-9.55; p=0.017, HR: 3.30, 95%CI: 1.78-10.52) were identified as independent factors associated with both RC and DSS (**Table 4**).

**Table 3:** Univariate analysis of predictors for regional control (RC) and disease specific survival (DSS) in cT1/2N0 maxillary squamous cell carcinoma.

Variable	p value for RC	p value for DSS
Age (≥50 vs <50)	0.398	0.442
Sex (Male vs female)	0.157	0.253
Smoker (Yes vs no)	0.877	0.564
Drinker (Yes vs no)	0.342	0.687
Tumor size (>2.0cm vs ≤2.0cm)	0.016	0.012
DOI^!^ (>5.0mm vs ≤5.0mm)	0.009	0.011
Midline involvement (Yes vs no)	0.142	0.203
PNI^ (Yes vs no)	0.028	0.135
LVI* (Yes vs no)	0.085	0.216
Pathologic grade (Poor vs intermediate vs well)	0.012	0.001
Neck management (SLNB vs conservative treatment)^&^	0.091	0.101

!DOI, depth of invasion; ^PNI, perineural invasion; *LVI, lymphovascular invasion; & SLNB, sentinel lymph node biopsy.

**Table 4:** Multivariable analysis of predictors for regional control (RC) and disease specific survival (DSS) in cT1/2N0 maxillary squamous cell carcinoma.

Variable	RC		DSS	
	p	HR [95%CI]	p	HR [95%CI]
Tumor size				
≤2.0cm		ref		ref
>2.0cm	0.014	2.63 [1.21-5.85]	0.005	3.04 [1.45-8.14]
DOI^!^				
≤5.0mm		ref		ref
>5.0mm	0.002	3.19 [1.62-9.87]	0.003	4.35 [1.58-10.69]
PNI^				
No		ref		
Yes	0.152	2.08 [0.76-4.96]		
Pathologic grade				
Well		ref		ref
Intermediate	0.211	2.87 [0.68-8.47]	0.368	3.21 [0.64-9.88]
Poor	0.024	3.16 [2.14-9.55]	0.017	3.30 [1.78-10.52]

!DOI, depth of invasion; ^PNI, perineural invasion; *LVI, lymphovascular invasion; &SLNB, sentinel lymph node biopsy.

### PSM

Tumor size, DOI, and pathologic grade were selected for PSM as they were identified as independent prognostic factors for RC and DSS. After a meticulous 1:1 matching process, a total of 80 patients were enrolled, with equal representation in both groups. Univariate survival analysis provided compelling evidence, demonstrating a statistically significant association between SLNB and improved rates of RC (p=0.033) but not DSS (p=0.129) when compared to observation (**Table 5**). These findings were further validated through an extensive Cox model, which confirmed the independent and protective efficacy of SLNB (p=0.021, HR: 0.72, 95%CI: 0.57-0.89) on RC (**Table 6**), indicating that patients who underwent SLNB had a 28% relative reduction in regional recurrence risk compared to those managed with observation alone.

**Table 5:** Univariate analysis of predictors for regional control (RC) and disease specific survival (DSS) in cT1/2N0 maxillary squamous cell carcinoma after propensity score matching.

Variable	p value for RC	p value for DSS
Age (≥50 vs <50)	0.364	0.227
Sex (Male vs female)	0.187	0.459
Smoker (Yes vs no)	0.511	0.378
Drinker (Yes vs no)	0.638	0.148
Tumor size (>2.0cm vs ≤2.0cm)	0.025	0.036
DOI^!^ (>5.0mm vs ≤5.0mm)	0.009	0.013
Midline involvement (Yes vs no)	0.691	0.855
PNI^ (Yes vs no)	0.334	0.285
LVI* (Yes vs no)	0.698	0.552
Pathologic grade (Poor vs intermediate vs well)	0.018	0.042
Neck management (SLNB vs conservative treatment)^&^	0.033	0.129

!DOI, depth of invasion; ^PNI, perineural invasion; *LVI, lymphovascular invasion; &SLNB, sentinel lymph node biopsy.

**Table 6:** Multivariate analysis of predictors for regional control (RC) and disease specific survival (DSS) in cT1/2N0 maxillary squamous cell carcinoma after propensity score matching.

	RC		DCC	
	p	HR [95%CI]	p	HR [95%CI]
Tumor size				
≤2.0cm		ref		ref
>2.0cm	0.015	2.42 [1.30-6.04]	0.002	2.42 [1.51-7.36]
DOI^!^				
≤5.0mm		ref		ref
>5.0mm	0.001	2.97 [1.47-7.58]	0.015	3.20 [1.63-8.11]
Pathologic grade				
Well		ref		ref
Intermediate	0.168	1.99 [0.74-6.85]	0.263	2.54 [0.78-8.13]
Poor	0.018	2.84 [1.54-7.32]	0.025	3.15 [1.54-9.38]
Neck management				
Conservative treatment		ref		
SLNB^&^	0.021	0.72 [0.57-0.89]		

!DOI, depth of invasion; &SLNB, sentinel lymph node biopsy.

### Subgroup analysis

**Figure 3:**
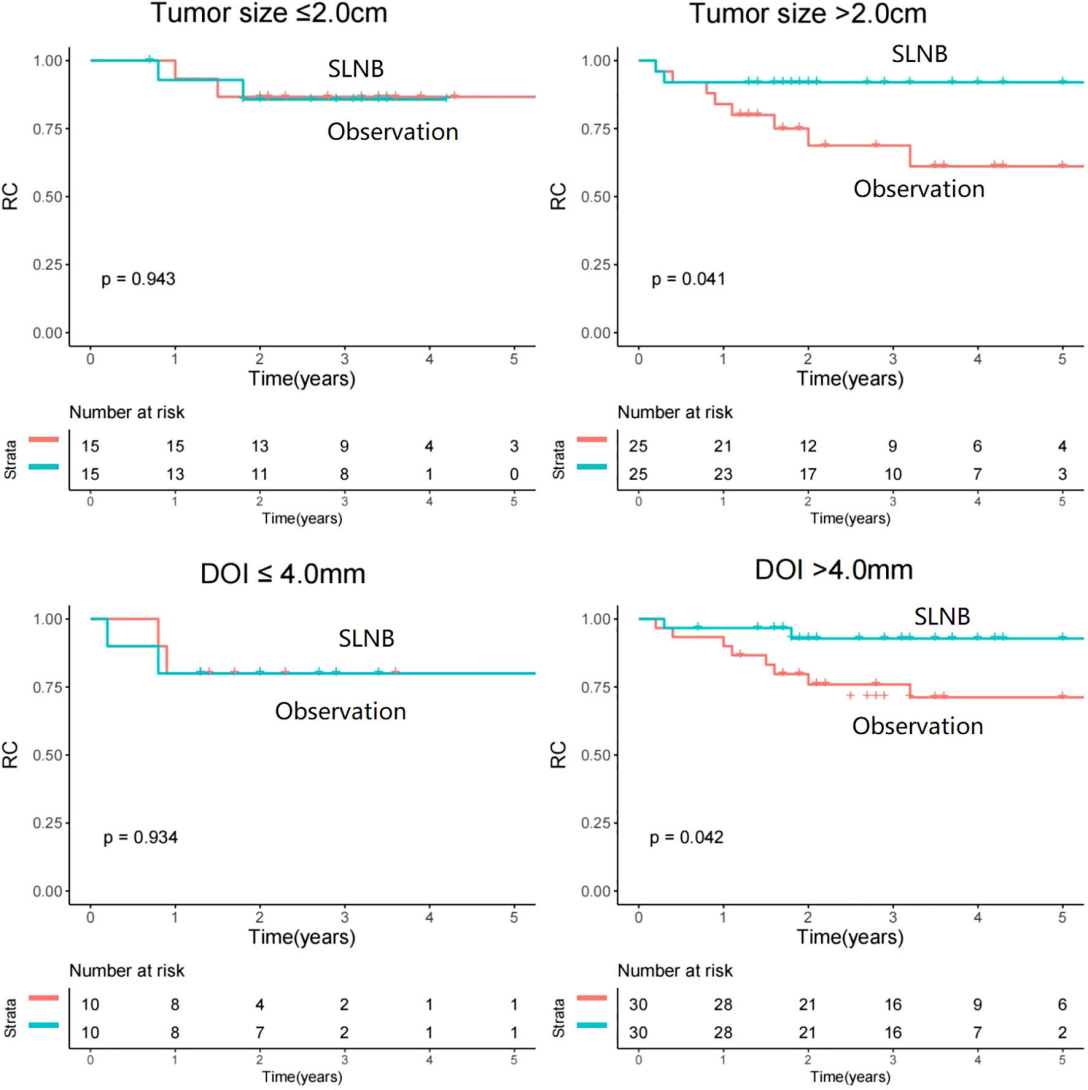
Comparison of regional control between sentinel lymph node biopsy (SLNB) and observation stratificated by tumor size and depth of invasion (DOI).

Among patients with tumor size ≤2.0cm, the use of SLNB displayed comparable rates of RC when contrasted with observation (p=0.943, HR=1.07, 95%CI: 0.15-7.63). However, an intriguing distinction emerged within patients with tumor size>2.0cm, wherein SLNB exhibited a significantly improved outcome in terms of RC (p=0.041, HR=0.23, 95%CI: 0.05-0.98) (**Figure 3**).

An investigation of a cutoff value of 4.0mm for DOI was conducted to help researchers correctly identify the subset of patients most likely to benefit from SLNB. Among patients with a DOI of ≤ 4.0mm, the rate of RC was similar between the SLNB and observation groups (p=0.934, HR=1.09, 95%CI: 0.15-7.71). However, in patients with a DOI of >4.0mm, SLNB was found to be a statistically significant contributor to improved RC (p=0.042, HR=0.22, 95%CI: 0.02-0.95) (**Figure 2**).

## Discussion

Our most important discovery revealed that when confronted with tumor size >2.0cm or a DOI exceeding 4.0mm in cT1/2N0 maxillary SCC, SLNB emerged as a compelling determinant of improved prognosis, surpassing the efficacy of mere observation in terms of RC. The study offered valuable insights into the potential candidates for SLNB or for observation alone. By identifying which patients were most likely to benefit from SLNB, clinicians could optimize treatment plans and provide more personalized care.

Maxillary SCC was relatively less common compared to SCC in other parts of the oral cavity, accounting for approximately 5% of all oral cancers (17). For a long time, it was believed to have limited LN metastasis potential, particularly in cases of cT1/2 disease. However, a recent review indicated that the overall rate of occult metastasis was 9.5% (range: 0-25%) for T1 maxillary SCC and 15.8% (range: 0-31.6%) for T2 tumors (18). This suggested that maxillary SCC might exhibited similar biological behavior to SCC in other oral sites. Consequently, the role of SLNB in these rare types of tumors became an urgent issue that required clarification. Our study was the first to investigate the question and found that SLNB was a highly effective technique, with a 100% detection rate of sentinel LNs. This finding is consistent with other reports that demonstrated the effectiveness of SLNB in identifying metastatic LN for SCC in other oral sites (6–8).

Distribution of sentinel LN location is important for clear decision of neck management. In theory, in cases where the tumor was situated in the upper gingiva, cancer cells had been postulated to disseminate to the submandibular LN via the buccal lymphatic system. However, when the primary tumor originated in the palate, cancer cells had the potential to travel to the deep cervical LNs through either the parapharyngeal or retropharyngeal lymphatic systems (19). To the best of our knowledge, the exploration of the lymphatic drainage pattern of sentinel LN in maxillary SCC had been seldom assessed. Boeve et al. (20) might had been the sole researchers to shed light on this matter, as they successfully detected and harvested sentinel LNs at cervical levels I, II, or III in their cohort of 11 patients—a finding that aligned with our own observations. Nonetheless, there existed a discrepancy between their study and ours, as they reported the detection of parapharyngeal sentinel LN in two patients, which was not observed in our investigation. This divergence could be attributed to the fact that our study exclusively enrolled patients with cT1/2 tumors, whereas the majority of their subjects presented with more advanced stages of disease. In such advanced cases, involvement of the soft palate may occur, with the parapharyngeal LN serving as a well-known metastatic site for oropharyngeal SCC.

Within the entire population analyzed, the overall rate of occult metastasis stood at 20.7%, a finding that aligned with previous reports (21). This observation suggested that the risk of LN metastasis in early-stage maxillary SCC was higher than initially anticipated, emphasizing the need for active intervention rather than mere observation in cases of cT1/2N0 maxillary SCC. Notably, our study might be the first to address this query and has revealed the superior prognosis provided by SLNB compared to observation alone. Such a finding was not unexpected, considering that LN metastasis often served as a critical prognostic factor. SLNB excelled in the timely detection of occult metastasis and accurate staging, rendering it a valuable tool to guide the administration of additional adjuvant therapies in the presence of a positive result. A singular team had previously explored the comparison between SLNB and observation (22). In that study, among the patients assigned to observation, three out of eleven individuals (27%) experienced regional recurrence, necessitating neck dissection. Contrastingly, in the SLNB group, only one out of eleven patients (9.1%) encountered regional recurrence. Although the difference did not reach statistical significance, there was a trend suggesting that SLNB was associated with improved RC. Some might argue that elective neck dissection could also be considered, as high-level evidence confirms its survival benefit in cT1/2N0 oral SCC. However, since maxillary SCC has a lower tendency for LN metastasis compared to SCC at other oral subsites, the risks of overtreatment and potential neck dysfunction should not be overlooked. In such cases, SLNB may serve as a viable alternative to neck dissection, offering comparable oncologic outcomes while preserving better quality of life (23, 24). Interestingly, we discerned that SLNB did not confer a survival advantage in DSS. This might reflect the efficacy of salvage therapy for nodal recurrence in the observation group, the limited sample size/follow-up to detect a survival difference, or the heterogeneity in radiotherapy technology and equipment attributable to the extensive temporal span of our study.

Since occult metastasis was primarily determined by tumor size and DOI, we evaluated whether these factors influenced the superior regional control achieved with SLNB compared to observation. Interestingly, SLNB was related to better prognosis in patients with tumors > 2.0cm versus ≤2.0cm; this differential effect may be attributed to the distinct biological behavior of smaller tumors (≤2 cm), which predominantly fall into the T1 category. Tumors in this size range exhibit a well-documented low propensity for lymph node metastasis, with reported occult metastasis rates of <10-15% (21). Consequently, the absolute benefit of SLNB is inherently limited in this subgroup due to their favorable natural history. In contrast, tumors >2 cm (typically T2 or greater) show significantly higher rates of nodal involvement, making nodal staging through SLNB clinically impactful for guiding subsequent management decisions. On the other hand, the protective effect of SLNB was not evident until a DOI of greater than 4.0mm. This observation could be attributed to the correlation between the capacity for LN metastasis and DOI. Notably, the incidence of regional recurrence was 5.2% for cases with a DOI less than 4.0mm, while it significantly escalated to 24.1% for those with a DOI equal to or greater than 4.0mm (25). The finding of our study was also supported by the NCCN guidelines, which recommended neck dissection in the presence of a DOI greater than 4.0mm. If the DOI was smaller than 4.0mm, the decision for neck dissection should be based on a discussion of the patient’s willingness and tumor characteristics.

PNI is a well-established prognostic factor in oral SCC, historically associated with poorer outcomes (26). However, our study found that PNI adversely impacted RC but not DSS. This discrepancy may be explained by the fact that PNI primarily reflects local tumor aggressiveness, promoting cancer spread along neural pathways and increasing the risk of locoregional recurrence. Importantly, since patients with PNI in our cohort typically received adjuvant therapy, the aggressive local management may have mitigated its effect on DSS, preventing a significant survival difference.

It is important to acknowledge the limitations in the current study. Firstly, as a retrospective study, there exists an intrinsic risk of selection bias in both the data collection and analysis processes, as well as in the variability of adjuvant radiotherapy protocols. Secondly, our comparatively modest sample size and the brevity of the follow-up period may have precluded the identification of any significant differences in DSS, thereby necessitating the undertaking of a multicenter study. Lastly, before the clinical application of our results, external validation is necessary to ensure the reliability and reproducibility of our findings.

## Conclusion

In conclusion, SLNB demonstrated noteworthy advancements in prognosis compared to observation alone in patients diagnosed with cT1/2N0 maxillary SCC. The protective effect of SLNB was particularly conspicuous in individuals presenting with tumor sizes exceeding 2.0cm or a DOI surpassing 4.0mm.

## Data Availability

The original contributions presented in the study are included in the article/supplementary material. Further inquiries can be directed to the corresponding author.
